# Comprehensive phenotyping of neuropsychiatric traits in a multiplex 3q29 deletion family: a case report

**DOI:** 10.1186/s12888-020-02598-w

**Published:** 2020-04-22

**Authors:** Melissa M. Murphy, T. Lindsey Burrell, Joseph F. Cubells, Michael T. Epstein, Roberto Espana, Michael J. Gambello, Katrina Goines, Cheryl Klaiman, Sookyong Koh, Rossana Sanchez Russo, Celine A. Saulnier, Elaine Walker, The Emory 3q29 Project, Hallie Averbach, Gary J. Bassell, Shanthi Cambala, Grace Carlock, Tamara Caspary, David Cutler, Paul A. Dawson, Michael P. Epstein, Henry R. Johnston, Elizabeth J. Leslie, Longchuan Li, Bryan Mak, Tamika Malone, Trenell Mosely, Rebecca M. Pollak, Ryan Purcell, Timothy Rutkowski, Jason Schroeder, Esra Sefik, Brittney Sholar, Nikisha Sisodoya, Sarah Schultz, Steven Sloan, Stephen T. Warren, David Weinshenker, Zhexing Wen, Michael E. Zwick, Jennifer Gladys Mulle

**Affiliations:** 1 Department of Human Genetics, Emory University School of Medicine, Whitehead 305M, 615 Michael Street, Atlanta, GA 30322 USA; 2 Department of Pediatrics, Emory University School of Medicine, Atlanta, USA; 3 Marcus Autism Center, Children’s Healthcare of Atlanta and Emory University School of Medicine, Atlanta, USA; 4 Departments of Human Genetics and Psychiatry and Behavioral Science, Emory University School of Medicine, Atlanta, USA; 5 Department of Psychiatry and Behavioral Science, Emory University School of Medicine, Atlanta, USA; 6 Department of Psychology, Emory University, Atlanta, USA; 7 Neurodevelopmental Assessment & Consulting Services, Decatur, USA; 8 Department of Epidemiology, Rollins School of Public Health, Emory University, Atlanta, USA

**Keywords:** 3q29 deletion, Schizophrenia, Psychosis, ADHD, CNV disorder, Evaluation of genetic syndromes, Case Report

## Abstract

**Background:**

3q29 deletion syndrome is associated with a range of medical, neurodevelopmental, and psychiatric phenotypes. The deletion is usually de novo but cases have been reported where the deletion is inherited from apparently unaffected parents. The presence of these unaffected or mildly affected individuals suggests there may be an ascertainment bias for severely affected cases of 3q29 deletion syndrome, thus the more deleterious consequence of the 3q29 deletion may be overestimated. However, a substantial fraction of 3q29 deletion syndrome morbidity is due to psychiatric illness. In many case reports, probands and transmitting parents are not systematically evaluated for psychiatric traits. Here we report results from a systematic phenotyping protocol for neurodevelopmental and neuropsychiatric traits applied to all 3q29 deletion carriers in a multiplex family.

**Case presentation:**

Through the 3q29 registry at Emory University, a multiplex family was identified where three offspring had a paternally inherited 3q29 deletion. We evaluated all 4 3q29 deletion family members using our previously described standardized, systematic phenotyping protocol. The transmitting parent reported no psychiatric history, however upon evaluation he was discovered to meet criteria for multiple psychiatric diagnoses including previously undiagnosed schizoaffective disorder. All four 3q29 deletion individuals in the pedigree had multiple psychiatric diagnoses that interfered with quality of life and prohibited successful academic and occupational functioning. Cognitive ability for all individuals was average or below average, but within the normal range.

**Conclusions:**

This is the first case report of inherited 3q29 deletion syndrome where all affected individuals in the pedigree have been comprehensively and systematically evaluated for neurodevelopmental and psychiatric symptoms, using a standard battery of normed instruments administered by expert clinicians. Our investigation reveals that individuals with 3q29 deletion syndrome may have psychiatric morbidity that is debilitating, but only apparent through specialized evaluation by an expert. In the absence of appropriate evaluation, individuals with 3q29 deletion syndrome may suffer from psychiatric illness but lack avenues for access to care. The individuals evaluated here all have cognition in the normal range alongside multiple psychiatric diagnoses each, suggesting that cognitive ability alone is not a representative proxy for 3q29 deletion-associated disability. These results require replication in a larger cohort of individuals with 3q29 deletion syndrome.

## Background

Individuals with 3q29 deletion syndrome are hemizygous for a 1.6 Mb interval containing 21 protein coding genes [1]. The syndrome is associated with a range of physical abnormalities, including heart defects, ocular abnormalities, and recurrent ear infections [2, 3]. Recent reports find that individuals with 3q29 deletion syndrome have increased susceptibility for neurodevelopmental and neuropsychiatric phenotypes, including mild to moderate intellectual disability, autism spectrum disorder (ASD), generalized anxiety disorder, and a remarkable 40-fold increased risk for schizophrenia [3–8]. The syndrome has most often been described in case reports, and these data have shaped our current understanding of the syndrome [1, 2, 9–27]. However, case reports often describe only physical exam results and do not include comprehensive neuropsychiatric evaluation with gold-standard instruments. Systematic descriptions of the neurodevelopmental and psychiatric phenotypes are emerging, but these have relied upon self-report of phenotypes [3, 7]. A comprehensive, unbiased characterization of the syndrome is needed.

While the syndrome is most frequently de novo, 20–30% of cases are inherited [28]. At least eight multiplex families have been reported in the literature, and the parent from whom the deletion is inherited is most often described as “apparently unaffected” or “mildly affected” [1, 13, 15–19]. These descriptions fuel suspicion that there are individuals in the general population who have the 3q29 deletion but manifest few of the phenotypic consequences. Mildly affected individuals are less likely to be referred for genetic testing, creating an ascertainment bias in favor of the most clinically overt cases rising to medical attention. This bias may result in overestimation of the disability associated with the 3q29 deletion phenotype.

However, in reports of multiplex families identifying these “mildly affected” or “apparently unaffected” individuals, the transmitting parent is typically evaluated with only a physical examination (Table 1) [1, 13, 15–19]. Neurodevelopmental and neuropsychiatric phenotypes have not been directly evaluated. These data may be extrapolated from the medical history and usually focus on the sole dimension of cognitive ability. As our appreciation of the myriad neuropsychiatric manifestations associated with 3q29 deletion syndrome has increased over time, it is important to reevaluate whether these transmitting parents are truly unaffected.

**Table 1:** Prior reports of 3q29 deletion multiplex families: summary of evidence for “unaffected” status of transmitting parents

First author (year)	Number of 3q29 deletion individuals in pedigree	Evaluation of proband	Evaluation of transmitting parent
Ballif (2008) [1]	3	No direct evaluation	No direct evaluation
Monfort (2008) [18]	2	Physical exam	Physical exam
Li (2009) [17]	2	Physical exam	Physical exam
Digilio (2009) [15]: Family #1	2	Physical exam, cognitive evaluation	Physical exam
Digilio (2009) [15]: Family #2	2	Physical exam	Physical exam
Clayton-Smith (2010) [13]	4	Proband: Physical exam Older brother: Physical exam, cognitive evaluation	Transmitting parent: Physical exam Transmitting grandparent: Physical exam
Petrin (2011) [19]	2	Physical exam	No evaluation^a^
Kahn (2019) [16]	2	Physical exam	Physical exam

^a^Transmitting parent reported in Petrin et al. [19] was mosaic for the 3q29 deletion

Through the 3q29 registry, we ascertained a multiplex family with three children, all with a paternally inherited 3q29 deletion. We evaluated all four affected individuals for neuropsychiatric traits, using normed, gold-standard instruments as part of our previously described comprehensive phenotyping protocol [29]. These data show that even within a single family there can be wide-ranging heterogeneity of phenotypes, and these psychiatric phenotypes may go undetected without focused evaluation by appropriate diagnosticians. Our data also reveal that in 3q29 deletion syndrome, individuals may exhibit substantial neuropsychiatric morbidity even when cognitive function is preserved. We conclude that periodic screening for neuropsychiatric illness should be prioritized for all individuals with 3q29 deletion syndrome, without regard for the presence of intellectual disability.

## Methods

**Fig. 1:**
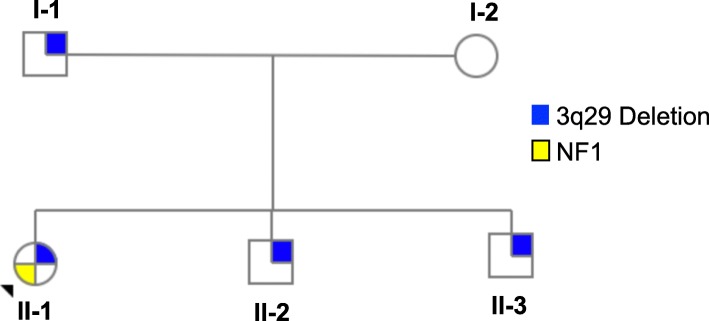
The 3q29 deletion multiplex family; individuals I-1, II-1, II-2, and II-3 were all evaluated in this study

A 4-person multiplex family (Fig. 1) was ascertained through the 3q29 registry (3q29.org [3], housed and maintained at Emory University), and enrolled in our ongoing research study. This study was approved by Emory University’s IRB (#IRB00088012). After an informed consent process with the parents was conducted by phone, we arranged the study visit. Medical records were obtained to confirm the genetic diagnosis. Informed consent was repeated in person at the beginning of the visit. Assent was also obtained from each of the children. Each family member was evaluated using our standardized and comprehensive phenotyping protocol with gold-standard instruments (Table 2, [29]). Study subjects were evaluated for cognitive ability [40, 41], anxiety [31, 37], executive function [33, 34], adaptive behavior [35], graphomotor ability [36], social disability [38, 39], psychosis spectrum symptoms [31, 32], and general psychopathology [30, 31]. All study subjects also had a medical history interview and physical exam conducted by an experienced medical geneticist. Instruments were scored according to established guidelines. Diagnostic cutoffs were determined based on published criteria; expert clinicians administered and scored all instruments and interpreted the scores to arrive at clinician best-estimate diagnoses. Quantitative scores were also extracted from each instrument. At the study visit, we obtained a blood sample from each family member. All blood samples were processed with an optical mapping technology (Bionano Genomics, San Diego CA) to confirm coordinates of the 3q29 deletion. Each family member was confirmed to have the canonical 1.6 Mb 3q29 deletion with identical breakpoints chr3:195998740–197,667,295 (hg38).

**Table 2:** Instruments and evaluations used in this study

Phenotype and Instrument	II-1 (Proband)	II-2 (8 yo male)	II-3 (4 yo male)	I-1 (39 yo male)
Medical history interview, Physical Exam	X	X	X	X
General psychopathology				
K-SADS [30]	X	X	X	
SCID-5-RV [31]				X
Prodrome/psychosis				
SIPS [32]	X	X	–	X
Executive function				
BRIEF-2 [33]	X	X	–	
BRIEF-A [34]				X
Adaptive behavior				
Vineland-3 [35]	X	X	X	X
Visual-motor integration				
Beery-Buktenica Developmental test of visual-motor integration, 6th ed. [36]	X	X	–	X
Anxiety				
ADIS-P, ADIS-C [37]	X	X	–	
SCID-5-RV [31]			–	X
Autism				
ADOS-2 [38]	Module 3	Module 3	Module 2	Module 4
ADI-R [39]	X	X	X	–
Cognitive Ability				
DAS-II, School Age [40]	X	X		
DAS-II, Early Years [40]			X	
WASI-II [41]				X

## Case presentations

### Individual II-1 (Proband): 9 yo female

#### History

The proband was a term 3.18 kg infant delivered by Caesarean section (C-section) due to breech presentation. Pregnancy was otherwise uncomplicated. The child exhibited a delay in her gross motor milestones, starting to walk around 2 years of age. Other milestones were met as expected. At age 4, the child started school and some behavioral difficulties were noted. Also emerging at this time were the presence of focal seizures, café-au-lait macules, and high blood pressure secondary to renal artery stenosis (treated surgically with a stent). A clinical diagnosis of neurofibromatosis 1 (NF1) is documented in her medical records by her pediatrician at age 4 years 7 months. Existing behavioral abnormalities in the proband were atypical for NF1 prompting a chromosome microarray that detected a 1.6 Mb 3q29 deletion. The clinical diagnosis of NF1 was confirmed by our medical geneticist (MJG) upon physical examination; thus this individual has both NF1 and 3q29 deletion syndrome. The child has poor weight gain due to food-related fussiness and retching. Current medications include two standard medications for blood pressure control, atenolol (beta blocker) and Lisinopril (ACE inhibitor); and carbamazepine, a standard anti-convulsant widely used for treating epilepsy. Of these medications only atenolol is not approved for use in children, though off-label use in pediatric cases is documented. At the time of our evaluation, the proband was receiving special education services for attention deficit hyperactivity disorder (ADHD).

#### Physical examination

Weight 20.5 kg (z = − 2.54) height 121 cm (z = − 2.29) frontal orbital circumference (FOC) 50.8 cm (35th percentile). Body mass index (BMI) was 14.0, 5th percentile. Blood pressure was 92/53. Mild dysmorphic features were present including a prominent forehead, low set posteriorly rotated, cupped ears, upslanting palpebral fissures, and asymmetric face with left eye higher than right. She had pes planus. There were multiple café au lait macules on the abdomen, chest and back, and axillary and inguinal freckling consistent with NF1.

#### Neuropsychiatric testing

This child was found to have a full scale IQ (FSIQ) of 82 (12th percentile), with verbal, nonverbal reasoning, and spatial reasoning subtest scores of 90, 74, and 91, respectively (noting a significantly lower nonverbal score). Her adaptive behavior (composite score = 65, 1st percentile) is lower than would be expected given her age and cognitive ability, indicating challenges with independent living skills. She also has clinically significant deficits in executive function (T-score 86, > 99th percentile). Her visual-motor integration score (standard score 92, 30th percentile) was within the average range, but her motor coordination subtest score (standard score = 67) revealed motor control deficits compared to below average visual perception (standard score = 82). Upon evaluation with the Kiddie Schedule for Affective Disorders and Schizophrenia (K-SADS), the child was found to meet criteria for ADHD, combined type. She does not exhibit anxiety, ASD (although social vulnerabilities were present), or psychosis-spectrum symptoms (ie, subclinical prodromal or psychotic symptoms).

### Individual II-2: 8 yo male

#### History

This child was a term 2.72 kg infant delivered by C-section due to a nuchal cord with no adverse sequelae. Delay of gross motor milestones was noted. He sat at 9 months and walked at 24 months and is yet to be completely toilet trained. Concerns about his behavior arose at age 3, concurrent with the time his older sister was diagnosed with 3q29 deletion syndrome. Genetic testing confirmed the presence of the 1.6 Mb 3q29 deletion in this child. Persistent concerns include inability to gain weight despite normal appetite, and abnormal sleep pattern of only ~ 3 h a night. No current or prior medication use was reported by the parent.

#### Physical examination

Weight 21.4 kg (z = − 1.58), height 126 cm (z = − 0.65), and FOC 49.5 cm (3rd percentile). BMI was 13.5, 1st percentile and underweight. Blood pressure was 106/75. Mild dysmorphic features were present including prominent forehead, frontal bossing, high anterior hairline, upslanted palpebral fissures, sparse eyebrows, wide nasal bridge and a dimpled chin.

#### Neuropsychiatric testing

This child was found to have a FSIQ of 79 (8th percentile), with verbal, nonverbal reasoning, and spatial reasoning subtest scores of 86, 80, and 79, respectively. His adaptive behavior score of 59 falls below the 1st percentile for his age and denotes significant delays in his adaptive functioning. He has clinically significant deficits in executive function (T-score = 88, > 99th percentile). Graphomotor weakness is present, indicated by a Visual-motor integration (VMI) standard score of 77, which falls at the 6th percentile for his age. On the VMI, he also showed significant motor coordination deficits (standard score = 45, < 1st percentile) compared to below average visual perception (standard score = 80, 6th percentile). Upon evaluation with the K-SADS, this child was found to qualify for diagnoses of ADHD, combined type; disruptive mood dysregulation disorder; and conduct disorder with childhood onset. Evaluation with the Anxiety Disorders Interview Schedule (ADIS) revealed diagnoses of Separation Anxiety and a Specific Phobia (fear of the dark). Evaluation with the SIPS revealed features consistent with Attenuated Psychosis Syndrome. No evidence of ASD was present but social vulnerabilities were noted.

### Individual II-3: 4 yo male

#### History

This child was a 2.27 kg term infant born by emergency C-section due to amniotic fluid loss. He had genetic testing at 6 months of age, after his sister, father, and brother were found to have the 3q29 deletion. He had delayed motor and verbal milestones; he walked at 24 months, and his first words were at 18 months. He is currently not toilet trained. An inguinal hernia repair was performed at age 4. He is underweight despite a normal appetite, and is followed by a dietitian. No current or prior medication use was reported by the parent.

#### Physical examination

Weight 11.7 kg (z = − 4.52) and height 95.6 cm (z = − 2.91) FOC (48.75 cm, 10th percentile). BMI was 12.8, < 1st percentile and underweight. Blood pressure was 112/53. Mild dysmorphic features were noted including a prominent forehead, frontal bossing, a dimple chin and deep set eyes. He has poor dentition. He had translucent skin and pes planus. He is reported to need very little sleep.

#### Neuropsychiatric testing

FSIQ was measured at 96 (39th percentile), with verbal, nonverbal reasoning, and spatial reasoning subtest scores of 103, 98, and 91, respectively. His adaptive behavior score of 68 (2nd percentile) indicates significant delays in his adaptive functioning given his age and cognitive ability. Due to young age, executive function, visual-motor integration, prodrome/psychosis, and anxiety were not evaluated (instruments are not normed at age 4). Symptoms endorsed by his mother using the KSADS parent interview revealed that this child was found to qualify for diagnoses of ADHD, hyperactive type. No evidence of ASD was present.

### Individual I-1: 39 yo male

#### History

The father’s genetic testing was completed when he was 34 years of age, after his daughter (the proband) was diagnosed with 3q29 deletion syndrome. He reports that he had early learning challenges. After graduating high school, he completed 3 years of postsecondary education, but did not complete his degree. He has held several jobs in the past but is currently unemployed and living on governmental assistance. He is working on obtaining a driver’s license. His father and paternal grandmother are both reported to have schizophrenia; these individuals were not available for genetic testing nor direct evaluation by our team. To the best of individual I-1’s knowledge, neither have had genetic testing to know if they also share the 3q29 deletion. He reported no prior neuropsychiatric diagnoses. He reports having difficulty falling asleep. No current or prior prescription medication use was reported.

#### Physical examination

Weight 77.4 kg (z = 0.55) height 165 cm (z = − 1.65), and FOC 54.5 cm are all within normal limits. BMI was overweight at 28.4. Blood pressure was 120/77. He had a prominent forehead but no other dysmorphic features were noted.

#### Neuropsychiatric testing

This individual’s FSIQ is 94 (34th percentile), with verbal comprehension of 99 and perceptual reasoning of 90. His adaptive behavior score of 61 falls below the 1st percentile for his age and denotes significant delays in adaptive functioning compared to his intact cognitive ability. Evaluation with the Behavior Rating Inventory of Executive Function, Adult edition (BRIEF-A) indicated significant executive functioning impairments (T-score = 73, 97th percentile). Mild graphomotor weakness is present, indicated by a VMI standard score of 83, which falls at the 13th percentile for his age. Visual perception and motor coordination are similarly developed (subtest standard scores = 81 and 86 respectively). Evaluation with the Structured Clinical Interview for DSM-5, Research Version (SCID-5-RV) revealed symptoms consistent with diagnosis of a prior panic disorder, and social anxiety disorder. No autism was present but social sequelae to his other disabilities were significant. This individual also meets criteria for ADHD, inattentive type. Upon evaluation with the SCID-5-RV and the Structured Interview of Psychosis-risk Syndromes (SIPS), and an independent qualitative evaluation by our team psychiatrist (JFC), the study subject endorsed multiple symptoms consistent with psychosis, including bizarre beliefs and auditory and visual hallucinations. Based on the study subjects’ report, his unusual sensory experiences most often coincided with mood disturbances and was therefore consistent with a diagnosis of schizoaffective disorder.

## Discussion and conclusions

Our team has performed direct, systematic evaluation of physical, neurodevelopmental, and psychiatric phenotypes using gold-standard instruments in members of a multiplex family where four related individuals have the 3q29 deletion. Physical manifestations are moderate and include subtle facial dysmorphology, an inability to gain weight, sleep abnormalities, and mild musculoskeletal abnormalities (Table 3). In contrast, the burden of psychiatric illness among the 3q29 deletion carriers in the pedigree is substantial (Table 4). All members of the pedigree qualify for a diagnosis of ADHD and have significant delays in adaptive behavior that are far greater than expected given their level of cognitive functioning. In fact, individual I-1’s (proband’s father) adaptive deficits were evident in his inability to sustain a job and function independently in many aspects of his life. Three individuals who were evaluated have clinically significant deficits in executive function, and two children have deficits in motor coordination. In two individuals, anxiety disorders were present, and in these same two individuals, evidence of a psychotic disorder is present. Individual II-1 (the proband) had a prior diagnosis of ADHD but no other neurodevelopmental or psychiatric diagnoses were reported in any member of the family. These data provide further evidence that the disability associated with 3q29 deletion syndrome is predominantly in the neuropsychiatric domain. The high burden of mental illness associated with the 3q29 deletion is an impediment to quality of life and independent functional living, but may be undetected without deliberate and focused evaluation.

**Table 3:** Physical exam results (NE, not evaluated)

System	II-1 (Proband)	II-2 (8 yo male)	II-3 (4 yo male)	I-1 (39 yo male)	Reported Frequency in 3q29 deletion syndrome
Growth					Short stature: 24%^2^
Weight kg (%tile)	20.5 (3)	21.4 (10)	11.7 kg (< 3)	77.4	Microcephaly: 55%
Height cm (%tile)	121 (3)	126 (35)	95.6 (3)	165	
FOC cm (%tile)	50.76 (35)	49.5 (3)	48.75 (10)	54.5	
Facial dysmorphism	Mild Poor dentition	Mild Poor dentition	Mild Poor dentition	Mild	Dental conditions 66%^3^ Abnormal teeth: 20%^2^
Musculoskeletal abnormalities	Flat feet	None noted	Slight joint laxity, flat feet	None noted	Ligamentous laxity: 11%^2^
GI	Feeding problems, inability to gain weight, fussy eater Poor appetite	Good appetite but inability to gain weight	Good appetite but inability to gain weight	None noted Reflux	Feeding problems: 41%^3^
Heart defects	none	none	none	none	Heart Defects: 26%^3^
Skin	Café au lait spots (NF1)		Translucent skin	Psoriasis	Abnormal skin pigmentation: 14%^2^
Sleep	No difficulties reported	Sleeps only ~ 3 h/night	Needs little sleep	Difficulty falling asleep	NE
Other	-Focal epilepsy -Renal artery stenosis s/p stent/HTN -Right eye wanders -Gags often -Hypermobility	-Not completely toilet trained -Aversion to loud noises -Cold intolerance	-Not toilet trained -Inguinal hernia repair -Sensitive hearing (likes ear defenders) -Poor coordination -Heat/cold intolerance	-Migraines	-Seizures: 5%^3^

**Table 4:** Neurodevelopmental and neuropsychiatric morbidity (NE, not evaluated)

Phenotype	II-1 (Proband)	II-2 (8 yo male)	II-3 (4 yo male)	I-1 (39 yo male)	Reported Frequency in 3q29 deletion syndrome
**General psychopathology**	ADHD, combined type	ADHD, Combined type; Disruptive Mood Dysregulation Disorder; Conduct Disorder with Childhood Onset	ADHD, hyperactive type	ADHD, inattentive type	ADHD: NE
**Prodrome/psychosis**	–	Attenuated Psychosis Syndrome	NE	Schizoaffective disorder	Psychosis: 5%^3^
**Executive function** **T-score (%tile)**	Clinically Significant Deficits 86 (> 99)	Clinically Significant Deficits 88 (> 99)	NE	Clinically Significant Deficits 73 (97)	NE
**Adaptive behavior** **Standard Score (%tile)**	Significant Delays 65 (1)	Significant Delays 59 (< 1)	Significant Delays 68 (2)	Significant Delays 61 (< 1)	Global Devel Delay 41%^3^
**Visual-motor integration** **Standard Score (%tile)**	Average Range 92 (30)	Significant Delays 77 (6)	Low 83 (13)	Low 83 (13)	NE
**Anxiety**	–	Separation Anxiety; Specific Phobia of the Dark	NE	Panic disorder; social anxiety disorder	Anxiety: 19%^3^
**Autism**	–	–	–	–	Autism: 26%^3^
**Cognitive Ability** **Standard Score (%tile)**	Below Average 82 (12)	Low 79 (8)	Average 96 (39)	Average 94 (34)	Intellectual disability: 92%^2^

Prior reports of multiplex families where transmitting parents are reported as “unaffected” or “mildly affected” have had an outsized effect on our interpretation of the 3q29 deletion phenotypic spectrum, introducing skepticism about the burden of illness associated with the deletion [1, 13, 15–19]. The existence of unaffected transmitting parents implies that there are individuals with the 3q29 deletion in the general population who are functioning within the average or above average range. These higher functioning individuals are not typically ascertained through genetic testing, thus it has been suggested that only the most severe cases of 3q29 deletion syndrome are described in the literature [10]. The implication of prior multiplex case reports is that the severity of the 3q29 deletion phenotype is overestimated. However, a reexamination of these multiplex reports reveals that phenotypic evaluation of transmitting parents has been limited to physical traits only, with no formal evaluation of cognitive ability or psychiatric illness. Many of these multiplex reports date back 10 years or more, when the association between the 3q29 deletion and psychiatric illness was not appreciated. In light of new findings that the 3q29 deletion is associated with generalized anxiety disorder, social disability, and increased risk for both autism and schizophrenia [2–6], it is appropriate to reconsider prior multiplex reports. Without direct evaluation and deliberate solicitation of psychiatric phenotypes, it is not known whether the transmitting parents reported in past case reports are truly unaffected. We note that in the multiplex pedigree we report on here, if criteria from prior case reports were used, the transmitting father would also have been judged to be only mildly affected. It was only through direct evaluation with appropriate instruments, and consideration of his psychosocial functioning and history, that his degree of disability emerged. Our data suggest a competing hypothesis: the adverse manifestations of the 3q29 deletion phenotype may be underestimated, as many prior case reports may not have ascertained the full spectrum of neurodevelopmental and psychiatric phenotypes in individuals with the 3q29 deletion.

In early descriptions of 3q29 deletion syndrome, subjects were noted to have “mild to moderate intellectual disability.” Subsequent case reports have therefore typically focused on the dimension of cognitive ability. However, in at least one prior report there is a hint that 3q29 comorbidity may be uncoupled from intellectual function. Cobb et al. reported on a 6.75 year old male with a de novo 3q29 deletion, with full-scale IQ measured at 84, well within the normal range [10]. However, this child was noted to have features consistent with autism and ADHD, inattentive type. The phenotypic features of Cobb et al’s patient are consistent with the individuals reported here, where cognitive measures for all 3q29 deletion individuals in the pedigree are within the normal range, yet multiple comorbid psychiatric diagnoses are present in each individual. Taken together, these data imply that the psychiatric phenotypes observed in 3q29 deletion syndrome are partially independent of intellectual function, and further suggest that the measure of cognitive ability is not a useful proxy for overall behavioral disability in individuals with 3q29 deletion syndrome. A larger sample of well-phenotyped 3q29 deletion study subjects is required to confirm these findings.

3q29 deletion syndrome has a heterogeneous presentation with variable penetrance of medical, neurodevelopmental and neuropsychiatric phenotypes [2, 3, 7]. This has been seen in other genomic disorders, such as 22q11.2 deletion syndrome [42] and 16p11.2 deletion and duplication [43, 44]. Various hypotheses have been invoked to explain this heterogeneity, including the presence of individual rare or common genetic variants that may act as modifier loci [45], genetic background effects [46], and/or polygenic risk scores [47, 48]. In the family evaluated in the current study, there is concordance among multiple neurodevelopmental and neuropsychiatric phenotypes that are exhibited (ADHD, executive function, adaptive behavior, visual-motor deficits are present in all evaluated) or not present (no member of the pedigree was diagnosed with autism spectrum disorder), suggesting that either these phenotypes are less sensitive to modifying influences, or the possible modifiers (whether genetic or environmental) are present in all family members. In contrast medical phenotypes are more variable, as are measures of intellectual function. FSIQ ranges from low (79) to average (96). In addition, two members of the pedigree were found to have a psychotic disorder. Individual III-3 was only 4 years of age at the time of evaluation; at this age psychotic phenotypes cannot be reliably assessed. The proband (Individual II-1) does not exhibit psychotic features at this time, but has not moved through the age at risk. Future follow up of this family would be useful to assess concordance of psychotic phenotypes. The current data suggest that medical phenotypes, intellectual function, and psychotic manifestations may be more susceptible to modifying influences. A larger sample of well-phenotyped individuals with 3q29 deletion syndrome will be required to support or refute these observations.

In conclusion, we report a multiplex family where multiple individuals have the 3q29 deletion syndrome and varied neurodevelopmental and psychiatric manifestations. These data serve to expand upon the 3q29 deletion phenotype and suggest that disability associated with the syndrome is not restricted to or solely a consequence of intellectual deficit. Our data further suggest that care and management of individuals with the 3q29 deletion should include screening for neurodevelopmental and psychiatric traits at multiple timepoints across the lifespan.

## Data Availability

All data generated or analyzed during this study are included in Tables 2 and 3. Additional details are available from the corresponding author on reasonable request.

## Abbreviations

ADHD: Attention Deficit Hyperactivity Disorder
ADIS: Anxiety Disorders Interview Schedule
ASD: Autism Spectrum Disorder
BMI: Body Mass Index
BRIEF: Behavior Rating Inventory of Executive Function
CNV: Copy Number Variant
C-Section: Caesarian section
FOC: Frontal Orbital Circumference
FSIQ: Full Scale Intelligence Quotient
KSADS: Kiddie Schedule for Affective Disorders and Schizophrenia
NF1: Neurofibramatosis 1
SCID-5-RV: Structured Clinical Interview for DSM-5, Research Version
SIPS: Structured Interview of Psychosis-risk Syndromes
VMI: Visual-Motor Integration
